# Meta-optic accelerators for object classifiers

**DOI:** 10.1126/sciadv.abo6410

**Published:** 2022-07-27

**Authors:** Hanyu Zheng, Quan Liu, You Zhou, Ivan I. Kravchenko, Yuankai Huo, Jason Valentine

**Affiliations:** ^1^ Department of Electrical and Computer Engineering, Vanderbilt University, Nashville, TN 37212, USA.; ^2^ Department of Computer Science, Vanderbilt University, Nashville, TN 37212, USA.; ^3^ Interdisciplinary Materials Science Program, Vanderbilt University, Nashville, TN 37212, USA.; ^4^ Center for Nanophase Materials Sciences, Oak Ridge National Laboratory, Oak Ridge, TN 37830, USA.; ^5^ Department of Mechanical Engineering, Vanderbilt University, Nashville, TN 37212, USA.

## Abstract

Rapid advances in deep learning have led to paradigm shifts in a number of fields, from medical image analysis to autonomous systems. These advances, however, have resulted in digital neural networks with large computational requirements, resulting in high energy consumption and limitations in real-time decision-making when computation resources are limited. Here, we demonstrate a meta-optic–based neural network accelerator that can off-load computationally expensive convolution operations into high-speed and low-power optics. In this architecture, metasurfaces enable both spatial multiplexing and additional information channels, such as polarization, in object classification. End-to-end design is used to co-optimize the optical and digital systems, resulting in a robust classifier that achieves 93.1% accurate classification of handwriting digits and 93.8% accuracy in classifying both the digit and its polarization state. This approach could enable compact, high-speed, and low-power image and information processing systems for a wide range of applications in machine vision and artificial intelligence.

## Teaser

Meta-optics enable lower power and higher speed image classification by off-loading operations from the digital system.

## INTRODUCTION

Digital neural networks and the availability of large training datasets have allowed for rapid progress in the performance of machine-based tasks for a wide range of applications including image analysis (*1*, *2*), sound recognition (*3*, *4*), and natural language translation (*5*). The enhanced capability, however, come at a computational cost as increased complexity and accuracy, has necessitated the need for ever larger deep neural networks (DNNs) (*6*). The ever-increasing computational requirements of DNNs have resulted in unsustainable growth in energy consumption and restrictions in real-time decision-making when large computational systems are not available.

One alternative to DNNs is the use of optical processors that have the advantages of ultrafast processing times and low energy costs (*7*, *8*, *9*). These systems can be used as stand-alone processors or as front-end accelerators for digital systems. In either case, optical systems are most impactful when used for the linear matrix-vector multiplications (*10*, *11*) that comprise the convolution operations in DNNs. These operation are often the most computationally burdensome components typically comprising more than 90% of the required floating-point operations (FLOPs) in popular DNNs (*12*, *13*). There are both free-space (*14*, *15*, *16*) and chip-based (*17*, *18*) approaches to optical processors, but, in either case, the computational advantage is achieved via the massively parallel and low-power processing that is possible with optics. In the case of image analysis, free-space approaches are attractive as spatial multiplexing can be readily achieved (*19*, *20*, *21*) and the fact that an optical front-end can potentially be integrated directly with an imaging system (*22*, *23*).

The most traditional approach to free-space–based optical image processing is the use of 4*f* optical correlators where spatial filters (*24*, *25*, *26*, *27*), either passive or dynamic, are placed in the Fourier plane of a two-lens optical system. Recorded spatial features are then fed to a lightweight digital neural networks back-end for classification. An alternative approach is the use of diffractive neural networks that use cascaded diffractive elements as convolutional layers (*28*, *29*, *30*). Image classification is realized through redistribution of optical energy on the detector plane requiring minimal digital processing. The tradeoff is the need for several diffractive layers and coherent illumination, precluding use with ambient lighting. While these approaches have shown benefits in terms of processing speed and energy consumption, they necessitate enlarged imaging systems. Furthermore, none of these approaches use the additional information channels, such as polarization, that are available when using an optical front-end (*31*, *32*).

Here, we demonstrate the use of meta-optic–based optical accelerators that serve as the convolutional front-end for a hybrid image classification system. Spatial multiplexing is achieved by using a multichannel metalens for image duplication and a metasurface-based convolutional layer. This system has the advantage of being compact while the use of metasurfaces allows for additional information channels (*33*, *34*, *35*, *36*), in this case, polarization, to be accessed, enabling both image and polarization-based classification. The hybrid network uses end-to-end design such that the optical and digital components are co-optimized while also incorporating statistical noise, resulting in a robust classification network. We experimentally demonstrate the classification of the Modified National Institute of Standards and Technology (MNIST) dataset (*37*) with an accuracy of 93.1% and a 93.8% accurate classification of polarized MNIST digits. Because of the compact footprint, ease of integration with conventional imaging systems, and ability to access additional information channels, this type of system could find uses in high-dimensional imaging (*38*), information security (*39*), and machine vision (*40*).

## RESULTS

**Fig. 1.**
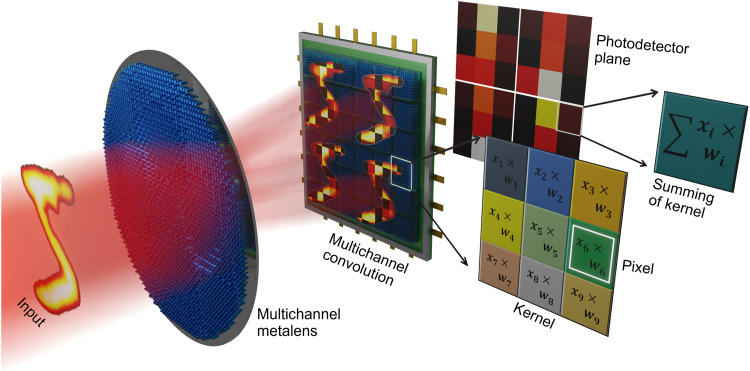
Schematic of the meta-optic accelerator. The meta-optic enables multichannel signal processing for replacing convolution operations in a digital neural network. Summing is achieved by each kernel being recorded by a single pixel on the photodetector.

**Fig. 2.**
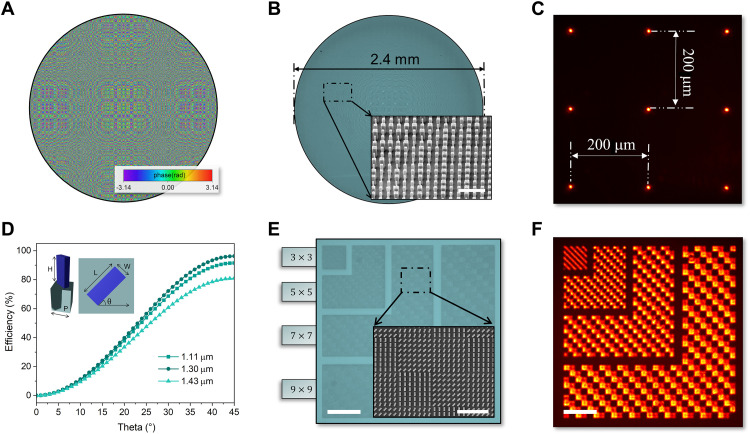
Components of the meta-optic system. (**A**) Phase profile of the multichannel metalens that was achieved using spatially multiplexed meta-atoms. (**B**) Optical image of the fabricated multichannel metalens. The inset is a scanning electron microscopy (SEM) image of the lens. Scale bar, 2 μm. (**C**) Experimental focal spot profile of the multichannel metalens under illumination at a wavelength of 1.3 μm. (**D**) Polarization conversion efficiency as a function of the meta-atom rotation angle. (**E**) Optical image of kernel resolution test chart. The left-side bar shows the number of meta-atoms in each kernel pixel. Inset is an SEM image of the meta-atoms. Left scale bar, 30 μm. Right scale bar, 4 μm. (**F**) Optical transmission of the kernel resolution test chart under tungsten lamp illumination. An orthogonal analyzer was used to image the transmission map. Scale bar, 30 μm.

The meta-optic accelerator is made up of two metasurfaces, a platform chosen because of the fact that it offers precise wavefront (*41*, *42*), complex amplitude (*43*), and polarization state (*44*) manipulation in an ultrathin form factor (*45*). Metasurfaces have also been used as standalone systems for all-optical image processing, namely, edge detection (*46*, *47*), through manipulation of the nonlocal, angle-dependent, optical response. In our design, the first metasurface is a multichannel metalens that duplicates an object into nine images as shown in Fig. 1. The multichannel metalens was created using nine meta-atoms per super cell to create images at nine spatial locations. The lens was created with a hyperbolic phase profile where the phase delay of each resonator, *i*, in the supercell is given by

$$\phi_{i}=\frac{2\pi }{\lambda }(f-\sqrt{f^{2}+{\left(x-a_{i}\right)}^{2}+{\left(y-b_{i}\right)}^{2}})$$ (1)

where *f* is the focal length, λ is the working wavelength, and *x* and *y* are the spatial positions on the lens. *a* and *b* correspond to the displacement of each unique focal spot, *i*, from the center of the lens. The resulting phase profile, for a 2.4-mm-diameter metalens, is shown in Fig. 2A. The metalens was realized using columnar silicon nanopillars with a period of 0.6 μm and a height of 0.88 μm. The transmission coefficient as a function of unit cell parameters can be found in section S1. The width of each meta-atom was chosen such it provides the phase profile given by Eq. 1. Fabrication of the metalens began with a silicon device layer on quartz patterned by standard electron beam lithography (EBL) and then followed by reactive ion etching (RIE). An optical image of the meta-lens is shown in Fig. 2B with the inset showing the individual meta-atoms. The experimentally recorded focal spots demonstrate diffraction-limited performance, as shown in Fig. 2C. While spatial multiplexing is used here to create the multichannel lens, it is worth noting that the design method is not unique. As additional channels are added, a spatially multiplexed lens will suffer from higher-order diffraction and resolution reduction due to a larger super cell structure. One way this can be overcome is through the use of complex-valued amplitude modulation that eliminates the need for spatial multiplexing. Using this technique, metalenses with multiple channels can be realized while preserving the spatial resolution of each image, as shown in section S2.

The second metasurface serves as a multifunctional kernel layer that provides the vector-matrix multiplication operations. The discretized kernels are based on Pancharatnam-Berry metasurfaces (*48*, *49*) that can encode polarization and/or amplitude information for convolution with the image. The transmission of each rectangular nanopillar comprising the metasurface follows an analytical model based on the Jones matrix given by

$$\left[\begin{array}{c} E_{x,\text{out}}\\ E_{y,\text{out}} \end{array}]=[\begin{array}{cc} \text{cos}(\theta ) & \text{sin}(\theta )\\ -\text{sin}(\theta ) & \text{cos}(\theta ) \end{array}][\begin{array}{cc} e^{i\phi_{x}} & 0\\ 0 & e^{i\phi_{y}} \end{array}][\begin{array}{cc} \text{cos}(\theta ) & -\text{sin}(\theta )\\ \text{sin}(\theta ) & \text{cos}(\theta ) \end{array}][\begin{array}{c} E_{x,\text{in}}\\ E_{y,\text{in}} \end{array}\right]$$ (2)

where *E*_*x*,in_, *E*_*y*,in_ and *E*_*x*, out_, *E*_*y*,out_ are the *x*- and *y*-polarized incident and transmitted amplitude, respectively. ϕ*_x_* and ϕ*_y_* are the phase shifts provided by the resonator for *x* and *y* polarization, values that are dictated by the size of the resonator. θ is the pillar rotation angle, which determines the polarization conversion efficiency for a given pixel in the metasurface. The kernel pattern is discretized to allow for a memory efficient architecture and one that is compatible with a dynamically reconfigurable system where pixelization is necessary due to practical limits on control electronics. To control the weights in each kernel, we use linearly polarized illumination combined with an orthogonal polarizer, serving as an analyzer, which is placed in front of the camera. The rotation angle of each meta-atom, θ, dictates the percentage, or weight, of the incident light that has had its polarization vector rotated by 90°, thus passing the analyzer. To achieve amplitude modulation, spatial variations in ϕ*_x_* and ϕ*_y_* are not needed and were fixed as ∣ϕ*_y_* − ϕ*_x_*∣ = π to simplify the model. In the case of *x*-polarized incident, light the intensity of *y*-polarized transmitted light is given by

$$I_{y,\text{out}}=\text{sin}{\left(2\theta \right)}^{2}∙I_{x,\text{in}}$$ (3)

where *I*_*x*,in_, *I*_*y*,in_ and *I*_*x*,out_, *I*_*y*,out_ are the *x*- and *y*-polarized incident and transmitted intensities, respectively. The use of pillar rotation for controlling kernel weight has the advantage of being broadband while also allowing for precise control over the weight as rotation is readily controlled in the lithography process. Figure 2D displays the transmission, *T_yx_* = *I*_*y*,out_/*I*_*x*,in_ as a function of rotation angle and wavelength, revealing a 320-nm bandwidth where there is less than a 10% variation in transmission. In this approach, either the camera pixel size or the kernel size determines the maximum areal density of neurons. In the case of the kernel, the meta-atoms in each pixel of the kernel are designed as being periodic. Thus, as the number of meta-atoms in each uniform pixel is reduced, there will be a deviation in the weight as the boundaries of the pixels, where periodicity is broken, play a larger role. In Fig. 2 (E and F), we characterize the role of pixel size on the accuracy of the designed weight using 3 × 3 pixel kernels and find that a minimum pixel size of 0.2 pixels/λ^2^ is possible based on a maximum weight error of 10%, where λ is the working wavelength. The quantitative analysis of kernel accuracy as a function of size can be found in section S3. Illumination at a wavelength of 1.3 μm yields a minimum pixel size of 3 μm by 3 μm (5 × 5 meta-atoms) or ~1 × 10^5^ pixels/mm^2^. This can be compared to state-of-the-art spatial light modulators (DLP650LNIR, Texas Instruments Inc.), where pixel sizes are on the order of 10.8 μm by 10.8 μm yielding 9 × 10^3^ pixels/mm^2^. Understanding the minimum metasurface pixel size is important for reconfigurable metasurface kernel layers as weight must be accurately controlled regardless of the kernel pattern.

**Fig. 3.**
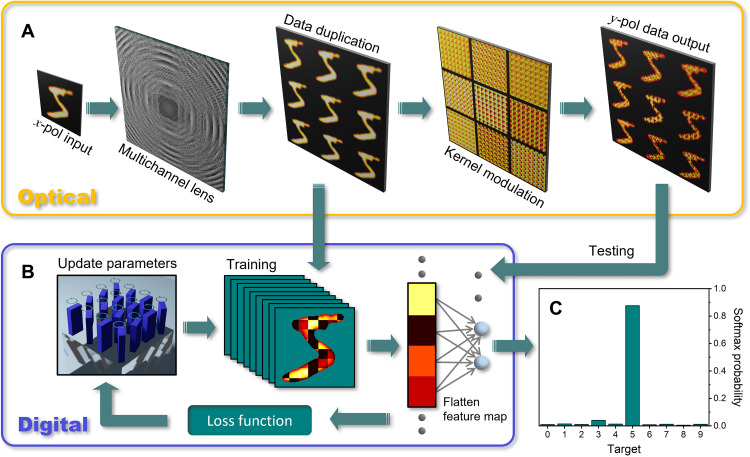
Workflow of the meta-optic accelerator design and testing process. (**A**) Metalens, with nine independent channels, splits the image. In training, these images are recorded on the camera and used for training of the optical kernel layer and digital systems. Once the kernel layer is trained, it is inserted into the system and the images are then projected onto that layer generating nine feature maps that are recorded on the camera (**B**) Digital loop comprises an iterative training process in which the Jones matrix is used for forward propagation. (**C**) Probability histogram is the final output for image classification.

To design the weights and geometry of the kernel layer, we use end-to-end design where both the digital and optical systems are co-optimized as shown in Fig. 3. The system was designed for classification of 24 × 24 pixel MNIST digits using the nine unique channels provided by the metalens, each channel comprising 3 × 3 pixel kernels with a stride of three. We use a shallow digital neural network comprising two fully connected layers with a rectified linear unit (ReLU) function in between. In this architecture, 50% of the overall FLOPs are implemented by the meta-optic accelerator. The detailed neural network architecture can be found in section S4. An optical front-end imposes unique constraints on the design of hybrid neural network structures as there are several noise sources in the analog signal being input, and output, from the optical system. The main sources of noise in our system come from stray light, detector noise, image misalignment due to variations in the optical system, aberrations from off-axis imaging system, and fabrication imperfections in the metalens and kernel layers. To better understand these noise sources and validate the designs for statistically relevant datasets, an image projection system was built for projecting the 24 × 24 pixel MNIST digits that comprised a spatial light modulation (SLM) illuminated with an incoherent tungsten filament lamp. The image characterization system is shown in section S5. The SLM was imaged, using the meta-optic, onto an InGaAs focal plane array that was triggered by the SLM such that large numbers of images could be recorded in an automated fashion.

To account for noise in the projection, imaging, and detector systems, the 10,000 training images from the MNIST dataset were projected and recorded using the metalens as the imaging optic, without the kernel layer, as shown in Fig. 3A. The optically recorded data were used as the training data in the end-to-end design loop. In the training process, we incorporated 10% spatial intensity fluctuation in the kernel layer and random image rotation within ±3°. The feature maps, which correspond to the convolution of the metasurface kernel layer with each of the nine images, were fed into the trainable model to form a mean-square-error loss function as shown in Fig. 3B. The backward propagation comprised a stochastic gradient descent–based algorithm driven by the loss function to update the physical parameters (ϕ*_x_*, ϕ*_y_*, and θ) of metasurface kernel layer for each iteration. The physical model, based on the Jones matrix, is used in forward propagation during design, and thus the evolution of the kernel weight is continuous with rotation angle, θ. Without the use of the physical model, one would have to clamp the weights in order for the transmission to be restricted to the physically attainable range of 0 to 1. Ultimately, the continuous weight evolution enabled by the physical model was found to result in more accurate classification by avoiding local minimum during training of the network (see discussions in section S6).

**Fig. 4.**
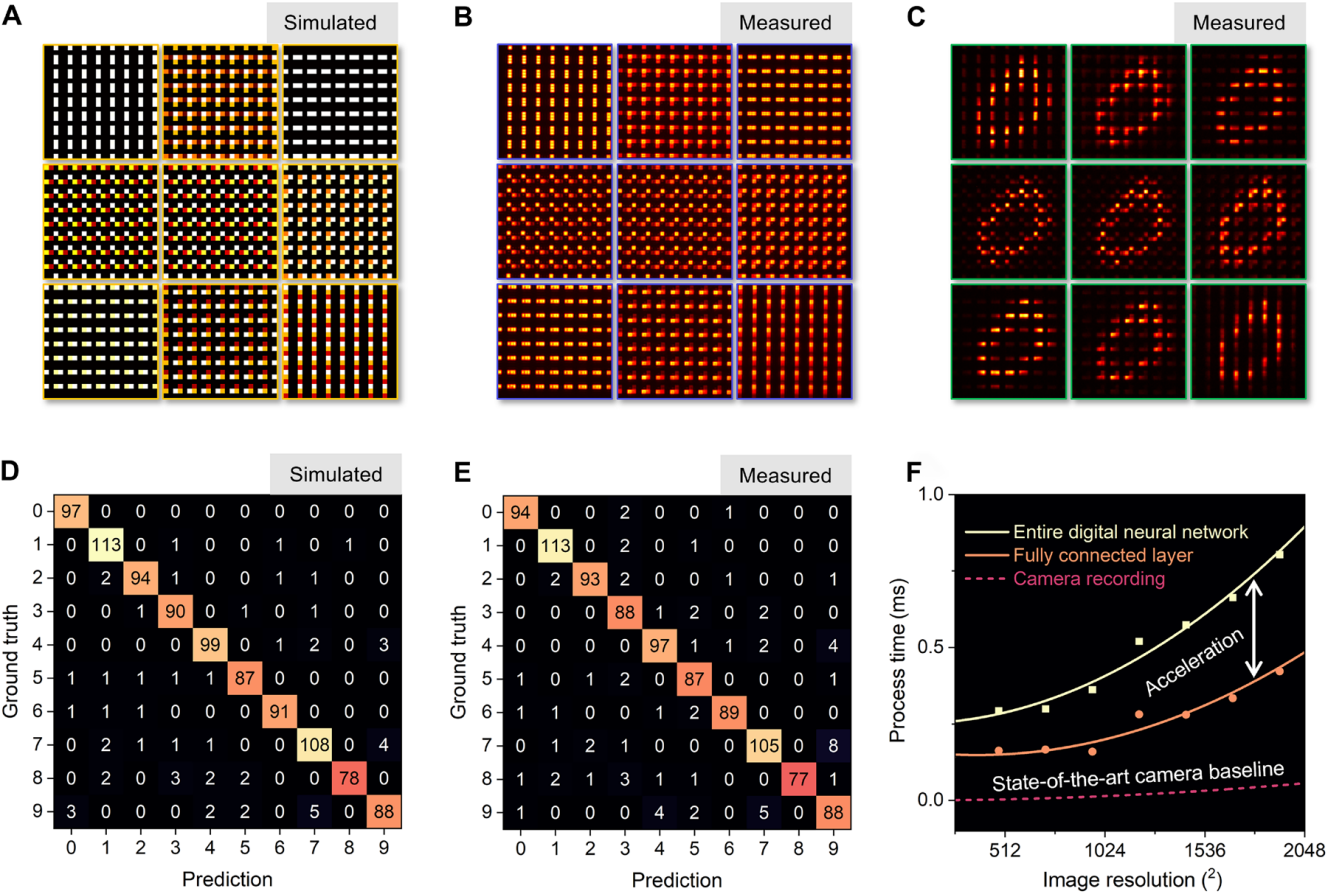
Classification of MNIST digits. (**A**) Transmission (weights) of the ideal kernels after the training process. (**B**) Measured transmission of the fabricated channels. An incoherent light source, filtered at 1.3 μm with a 30 nm bandwidth, was used for illumination. (**C**) Nine feature maps recorded on the camera. (**D** and **E**) Theoretical and measured confusion matrices for MNIST object classification, respectively. (**F**) Acceleration by optical convolution in neural networks as a function of input image resolution. The state-of-the-art camera baseline indicates the image recording speed by Phantom TMX 7510.

Once training of the system was complete, the metasurface kernel layer was realized by using a silicon film on quartz with the device layer patterned into nanopillars with a period of 0.6 μm and a height of 0.88 μm. The metasurface was fabricated using EBL patterning followed by RIE. The width and length of each nanopillar were fixed as 160 and 430 nm, respectively, with the rotation angle set through training of the hybrid network. The metasurface kernel layer was imaged using uniform illumination and compared to the theoretical design, both of which are included in Fig. 4 (A and B). The fabricated and designed kernels show agreement with an SD of less than 10%, which matches the noise level in the training model. The kernel layer was then placed in the image plane of the metalens for recording convoluted images from the testing dataset. Figure 4C shows the feature map produced for a digit of “0.” Each kernel pixel comprises an 11 × 11 (6.6 μm by 6.6 μm) meta-atom array. Summing of each kernel could be achieved optically via alignment of each kernel with an individual pixel on the camera; however, in this work, summing is performed digitally as the kernel layer is magnified when imaged onto the camera such that each kernel comprises multiple camera pixels.

To characterize the system’s performance, 1000 digits, not in the training set, were recorded using the meta-optic. The theoretical and experimental confusion matrices for this testing dataset are shown in Fig. 4 (D and E, respectively). The theoretical training model’s overall accuracy was 94.7%, while the experimental accuracy is 93.1%. To validate the significance of the optical convolution layer, two types of reference experiments were performed, one with no convolution layer and one with random kernel values, both based on the same neural network architecture as used with the meta-optic front-end. If the convolution layer is removed, then a dense neural network with comparable FLOPs was found to have 35.0% accuracy. If a random, untrained, kernel layer is used, then a model accuracy of 79.7% was obtained. Both benchmarks illustrate the significance of both the meta-optic front-end and end-to-end design (see details in section S7). Although this proof-of-concept demonstration involves low-resolution images, the small minimum pixel size of the kernel layer along with the parallel nature of the optical operations means that this architecture could be a powerful tool for high-speed and large-scale image processing applications. In Fig. 4F, we benchmark system performance based on both the all-digital and hybrid networks recording images with a state-of-the-art camera (Phantom TMX 7510) and the same network architecture and hardware platform. The hybrid network is accelerated because of the passive and parallel convolution operations provided by the metasurface with gains increasing with increase of image resolution. Moreover, the versatility of the system can be further improved by the incorporation of dynamically tunable metasurfaces (*50*) as the kernel layer such that the optical front-end can be reconfigured or temporally multiplexed.

The complexity of the neural network, described using FLOPs, dictates the robustness and complexity of the classification task. The stride number is related to the system complexity and, in the approach described here, is limited to the kernel size due to the spatial multiplexing method used. Although robustness was optimized during the training process, the complexity of the system could be further enhanced by reducing the stride number of the optical convolution operations. This can be achieved using complex-valued amplitude modulation in the kernel layer providing for control over image overlap and the ability to achieve arbitrary stride values. The detailed design process and experimental verification are shown in section S8.

**Fig. 5.**
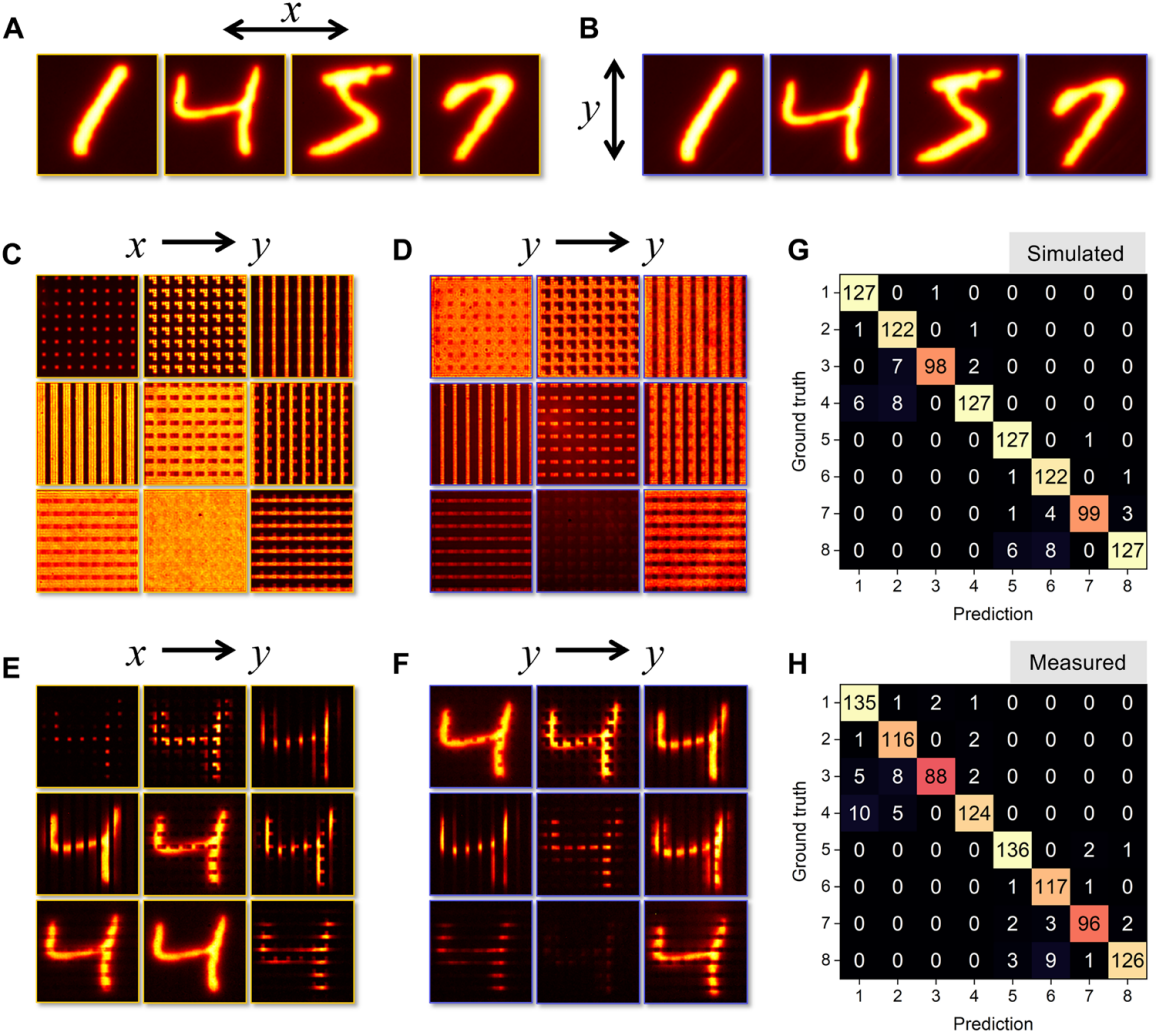
Demonstration of multifunctional object classification. (**A** and **B**) Digits for classification that carry both amplitude and polarization information. (**C** and **D**) Transmission profiles of the fabricated metasurface channels under incoherent, 1.3 μm wavelength, illumination with orthogonal linear polarization states. (**E** and **F**) Feature maps for identical digits with *x* and *y* polarization, respectively. (**G** and **H**) Theoretical and measured confusion matrices, respectively.

One of the unique strengths of metasurfaces, compared to conventional lenses or diffractive optical elements, is their ability to provide user-specified amplitude and phase functions while also being sensitive to the polarization state and wavelength of light (*51*). This allows for access to additional information carriers that are normally lost when recording an image on a camera enabling one to discriminate based on normally hidden features in the physical world such as vectorial polarization, phase gradients, or spectrally complex signals. To demonstrate this ability, a polarized MNIST dataset with 8000 images was created comprising four digits (1, 4, 5, and 7) with each digit having two orthogonal polarization states, as shown in Fig. 5A. More complex, nonorthogonal signal classification is also achievable using this approach (section S9). In this case, a single fully connected digital layer, without ReLU, was used for classification (see section S4). Polarization classification is possible due to the fact that the meta-atoms, outlined in Fig. 2D, have a transmitted intensity that is dependent on the incident polarization state, given by

$$I_{y,\text{out}}=\text{cos}{\left(2\theta \right)}^{2}∙I_{y,\text{in}}+\text{sin}{\left(2\theta \right)}^{2}∙I_{x,\text{in}}$$ (4)

where *I*_*x* in_, *I*_*y*in_, and *I*_*y*,out_ are the *x*- and *y*-polarized incident and transmitted intensities and θ is the meta-atom rotation angle. While the output signals between the *x*- and *y*-polarized channel are correlated, the mechanism for polarization recognition is independent from object classification and the later requires feature map analysis provided by the convolution process. In the case of polarization, the birefringence of the meta-atoms enables the conversion of the polarization state to an intensity value. The intensity arising from the polarization state is constant across the channel and is completely independent of the spatially varying amplitude value. Hence, both functions, polarization and amplitude classification, can be integrated into a single meta-optic for multifunctional analysis. The optical kernel layer was designed following the training procedure outlined in Fig. 3 with the system tasked with classifying eight output states comprising the four distinct digits, each with *x* and *y* polarization states. The metasurface kernel layer was formed from rectangular nanopillars using the same geometry and fabrication process as described previously. In Fig. 5 (C and D), the transmitted intensity, *I*_*y*,out_, is provided for a uniformly illuminated kernel layer with both *x* and *y* polarization states, and in Fig. 5 (E and F), we provide the feature maps for an identical digit with *x* and *y* polarization states. Both the uniformly illuminated kernels and feature maps demonstrate the contrast in the convolution for orthogonal polarization states. In Fig. 5 (G and H), we provide the theoretical and experimental confusion matrices, respectively, for 1000 test images not in the training dataset. The theoretical accuracy of classification was 94.8%, while the experimental accuracy was 93.8%, showing excellent agreement. It is worth mentioning that in the confusion matrix, the neural network system has a 100% accuracy on polarization state recognition.

## DISCUSSION

In conclusion, we have demonstrated a meta-optic accelerator for multifunctional image classification. The technique is enabled by the unique design freedom afforded by metasurfaces, including the creation of multichannel lenses to duplicate information and polarization-sensitive kernel layers that allow for discrimination based on both the spatial intensity profile and the polarization state of the object. The use of polarization demonstrates how optical front-ends are able to access additional information channels normally lost in traditional imaging systems. Furthermore, by implementing end-to-end design, we improved the robustness of the system to common noise sources ultimately yielding ~94% experimental classification accuracy that closely matches the theoretical prediction.

The proposed meta-optic accelerators can be massively parallel and serve to bridge the gap between the natural object and digital neural network analysis. The approach can allow one to harness the strengths of both free-space and electronic or optical chip–based architectures. Moreover, the ability to operate with incoherent illumination enables machine-vision applications with passive ambient lighting that is incompatible with diffractive neural networks. The current optical approach is limited to linear operations, which prevents the use of activation functions, but these types of layers could be added in the future based on nonlinear media. Even without optical activation functions, further optimization of the neural network architecture could be used to off-load more linear operations into the front-end. End-to-end optimization also provides a robust platform that can balance the trade-off between bandwidth and the aperture size for a meta-optic system (see the discussion in section S10). Ultimately, these advantages allow meta-optic accelerators to achieve superior processing speed while also lowering power consumption and thus could lead to advances in a wide range of compact, low-power, and high-speed computer vision systems.

## MATERIALS AND METHODS

### Digital neural network training

An end-to-end optimization process was used to design the digital neural network system for object classification. Training data were first generated by projecting, and imaging, the 10,000 training images from the MNIST data library. The nine optical images generated from the multichannel metalens were recorded at 512 × 640 resolution, and then each image was down-sampled to 24 × 24 pixels. During forward propagation in the neural network, 10% random intensity fluctuation and random image rotation within ±3° were added to improve the system robustness. In training of the digital and optical components of the network, an Adam optimizer was used with the learning rate set as 0.001. Training occurred over 50 epochs. The detailed neural network architecture is shown in section S4. All the algorithm was programmed on the basis of PyTorch 1.10.1 and CUDA 11.0 with Quadro RTX 5000/PCIe/SSE2 as the graphics card.

### Metasurface fabrication

EBL-based lithography was used to manufacture all of the metasurface layers. First, plasma-enhanced chemical vapor deposition was used to deposit an 880-nm-thick silicon device layer on a quartz substrate. Polymethyl methacrylate (PMMA) photoresist was spin-coated on the silicon layer followed by evaporation of a 10-nm-thick Cr conduction layer. EBL patterning was performed, and after removing the Cr layer, the exposed pattern was developed by methyl isobutyl ketone solution. A 50-nm Al_2_O_3_ hard mask was deposited via electron beam evaporation, followed by lift-off. The silicon was then patterned using RIE, and a 1-μm-thick layer of PMMA was spin-coated to encase the nanopillar structures as a protective and index-matching layer.

### Numerical simulations

The complex transmissive coefficient of the silicon nanopillars was obtained using an open-source rigorous coupled-wave analysis solver (RETICOLO) (*52*). A square lattice with a period of 0.6 μm was used with a design wavelength of 1.3 μm.

### Optical training and testing system

A commercial liquid crystal–based SLM (PLUTO-2.1 phase only, HOLOEYE) was used to create the optical images using illumination from an incoherent light source (66997-100Q-R085, Newport) with a band-pass filter (FB1300-30, Thorlabs). The SLM modulates the polarization state of the light, which is converted into amplitude modulation by using a polarizer and analyzer before and after the SLM. The multichannel metalens was used to project the image onto the kernel layer. The kernel layer was imaged onto the camera (Wildcat 640, Xenics) using a tube lens, and a polarizer was placed in front of the camera. A detailed schematic of this system is provided in section S5.

## Supplementary Materials

### This PDF file includes:

Sections S1 to S10 Figs. S1 to S10
