# Supplementary material for: Meta-optic accelerators for object classifiers
Source: Sci Adv. 2022 Jul 27;8(30):eabo6410. doi: 10.1126/sciadv.abo6410 (PMC9328681; doi:10.1126/sciadv.abo6410)
Supplement: Supplementary file 1 — Sections S1 to S10 Figs. S1 to S10 [file sciadv.abo6410_sm.pdf]

Science Advances
AAAS

Supplementary Materials for

Meta-optic accelerators for object classifiers

Hanyu Zheng *et al.*

Corresponding author: Jason Valentine, jason.g.valentine@vanderbilt.edu

Sci. Adv. **8**, eabo6410 (2022)
DOI: 10.1126/sciadv.abo6410

This PDF file includes**:**

Sections S1 to S10
Figs. S1 to S10

S1: Transmission coefficient and complex amplitude control using nanopillar meta-atoms

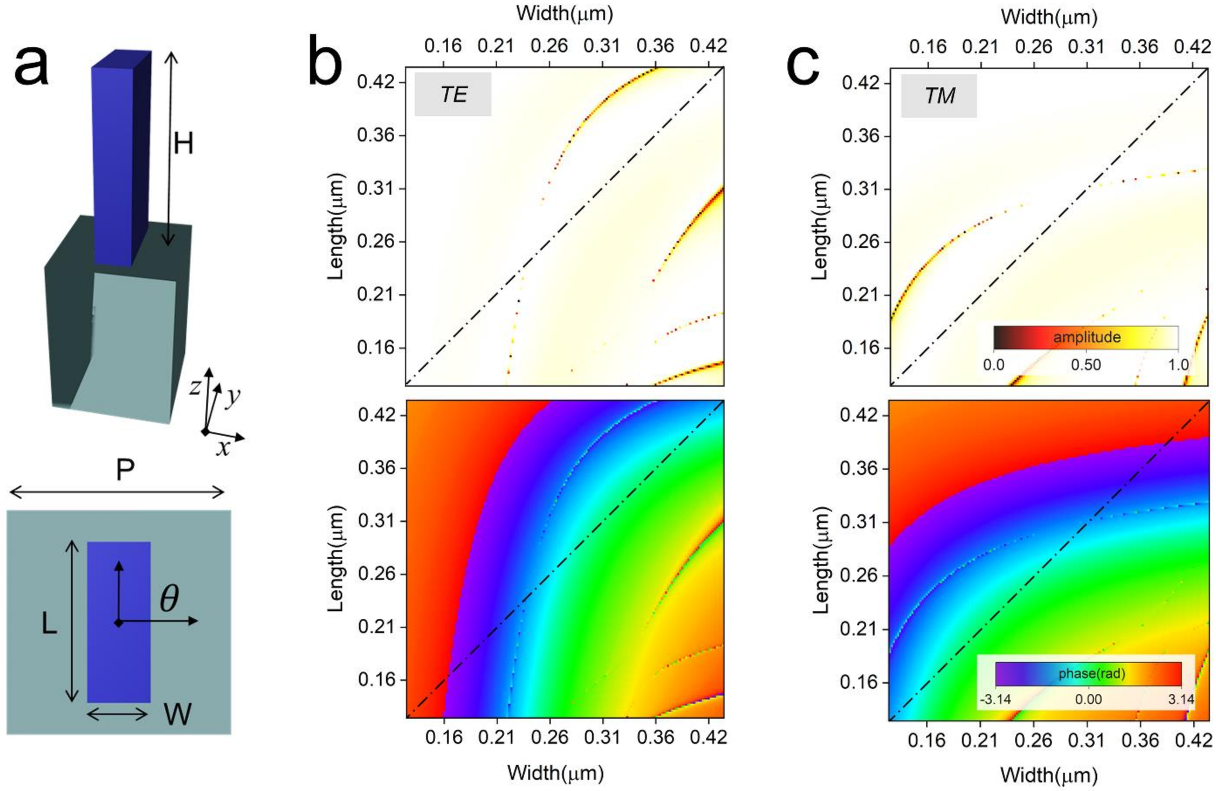

Figure S1. The simulation results of meta-atom used in the manuscript. (a) The schematics of birefringent meta-atom composed of silicon nanopillar on glass. The height was fixed at 880nm with period of 600nm at working wavelength of 1.3μm. θ is set as 0 to extract the fundamental mode response under TE and TM excitation. (b) The amplitude and phase profile as a function of width and length of silicon nanopillar. The input light is polarized as TE mode (along *x*-axis). (c) The amplitude and phase profile under TM mode (along *y*-axis) excitation.

The complex transmission coefficient of nanopillar meta-atoms was calculated using rigorous coupled wave analysis (RCWA) as shown in Fig.S1. The schematic of the unit cell is displayed in Fig.S1 (a). The height and period of the silicon structure were fixed at 880nm and 600nm, respectively, with the material index of 3.62 and an illumination wavelength of 1.3μm. Considering the subwavelength period, only the fundamental (zeroth) transmission mode (both TE and TM) was extracted from the simulation. During the parameter (width and length) scanning, the rotation angle (θ) was set as 0, which forms the birefringent optical response map shown in (b) and (c). The dash lines in (b) and (c) indicate the isotropic optical response (polarization insensitive), which was used for the multi-channel metalens design in the manuscript.

To achieve complex-amplitude modulation, the output phase ($0\sim2\pi$) and amplitude ($0\sim1$) of the meta-atom need to be controlled independently. The optical response of the meta-atoms can be described by Jones matrix,

$$\begin{bmatrix} E_{x,out} \\ E_{y,out} \end{bmatrix} = \begin{bmatrix} cos(\theta) & sin(\theta) \\ -sin(\theta) & cos(\theta) \end{bmatrix} \begin{bmatrix} e^{i\phi_x} & 0 \\ 0 & e^{i\phi_y} \end{bmatrix} \begin{bmatrix} cos(\theta) & -sin(\theta) \\ sin(\theta) & cos(\theta) \end{bmatrix} \begin{bmatrix} E_{x,in} \\ E_{y,in} \end{bmatrix} \quad \text{(S1)}$$

where $E_{x,in}$, $E_{y,in}$ and $E_{x,out}$, $E_{y,out}$ are the x and y polarized incident and transmitted amplitude. ϕ_x and ϕ_y are the phase shifts provided by the resonator for x and y polarization. θ is the pillar rotation angle. If we only consider x-polarized light input and y-polarized output, the transmission can be simplified as:

$$E_{y,out} = \left(e^{i\phi_y} - e^{i\phi_x}\right)sin(\theta)cos(\theta)E_{x,in} \quad \text{(S2)}$$

$$E_{y,out} = \frac{1}{2}e^{i\phi_x}\left[e^{i(\phi_y-\phi_x)} - 1\right]sin(2\theta)E_{x,in} \quad \text{(S3)}$$

If the condition $\phi_y - \phi_x = \pi$ is satisfied for arbitrary ϕ_x, then the final transmission is described by,

$$E_{y,out} = -e^{i\phi_x}sin(2\theta)E_{x,in} \quad \text{(S4)}$$

Eq.S4 demonstrates the ability to achieve complex-valued amplitude modulation, where the output phase and amplitude are dictated by ϕ_x and θ, respectively.

S2: Complex amplitude modulation for the multichannel metalens

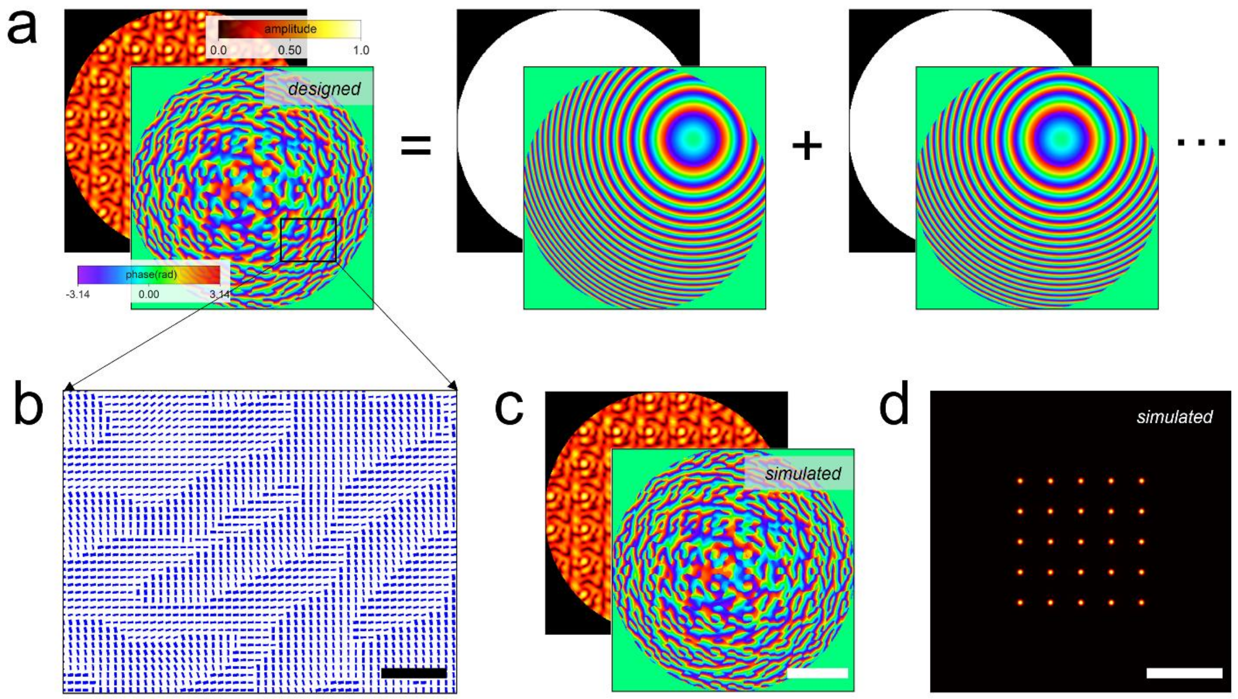

Figure S2. The complex-amplitude modulation method for multi-channel metalens. (a) The design process for multi-channel metalens with arbitrary information channels. (b) The schematics of metasurface profiles to construct the complex-amplitude profile in (a). Scale bar: 5μm. (c) The reconstructed complex-amplitude profile based on calculated meta-atoms' data library. Scale bar: 50μm. (d) The calculated focal spot profile of the multi-channel metalens based on complex-amplitude values in (c). Scale bar: 50μm.

In the manuscript, we demonstrated a 3×3 multi-channel metalens based on spatial multiplexing of the unit cells. In this case, higher diffraction orders will be problematic as the number of channels is increased. To create arbitrary numbers of channels while eliminating higher diffraction orders, complex-valued amplitude modulation of the output field is necessary.

A complex amplitude metalens with 25 output channels is exhibited in Fig.S2. The complex-valued field profile can be described by the following equation,

$$F[\alpha(x,y),\phi(x,y)] = \sum_i^n \exp\left[\ i \cdot \frac{2\pi}{\lambda}\left(f - \sqrt{f^2 + (x-a_i)^2 + (y-b_i)^2}\right)\right] \qquad \text{(S5)}$$

where f is the focal length, λ is the working wavelength, and x and y are the spatial positions on the lens. a and b correspond to the displacement of each unique focal spot, i, from the center of the lens. F is the complex-amplitude field composed of α (amplitude) and ϕ (phase) information. In Fig.S2, we present the design and simulation of a 5×5 multi-channel metalens based on this

method. The ideal complex-amplitude field for the multi-channel metalens is shown in Fig.S2 (a). To reconstruct this field, the nanopillars discussed in section S1 were employed and the required geometrical data for the metasurface is exhibited in Fig.S2 (b). The reconstructed complex-amplitude field, displayed in Fig.S2 (c), shows an excellent match with the designed target field. The focal spot profile was calculated based on the angular spectrum propagation method and is shown in Fig.S2 (d), demonstrating independent multi-channel imaging. The limitation of multiplexed channel number, in this case, is that the field of view is reduced as the number of channels increases. As with spatial multiplexing, multiple multi-channel lenses can be patterned to overcome this issue.

S3: Kernel resolution characterization

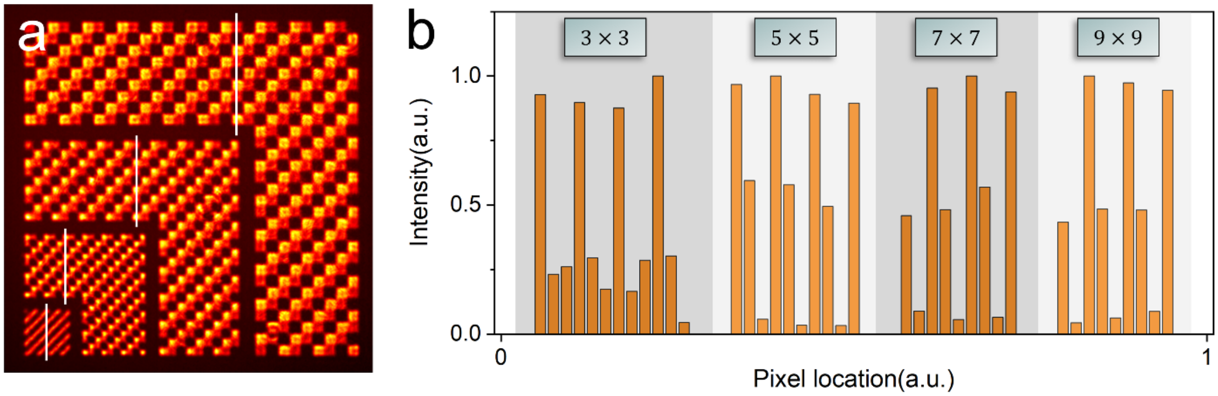

Figure S3. The characterization of metasurface-based kernel resolution. (a) The field profile of test kernel in manuscript. The white lines indicate the extracted data in. (b) The data was extracted according to different meta-atoms number in each kernel element. (c) The quantitative description of kernels as a function of meta-atoms number is each kernel element. The top labels indicate the meta-atoms arrangements in a single kernel pixel.

The kernel resolution was characterized based on a metasurface-based test chart, where a 3×3 kernel was formed with different numbers of meta-atoms in each kernel pixel. The kernel comprised weights of 0, 0.5 and 1 arranged in a checkboard layout as shown in Fig.S3 (a). Under the illumination of a tungsten lamp, the transmission was measured using a camera as shown in Fig.S3 (a). The weight of each kernel, measured along the white lines in Fig.S3 (a), is displayed in Fig.S3 (b). It was found that the metasurface weight error is less than 10% when the number of meta-atoms in each kernel is larger than 5×5.

S4: Neural network architecture

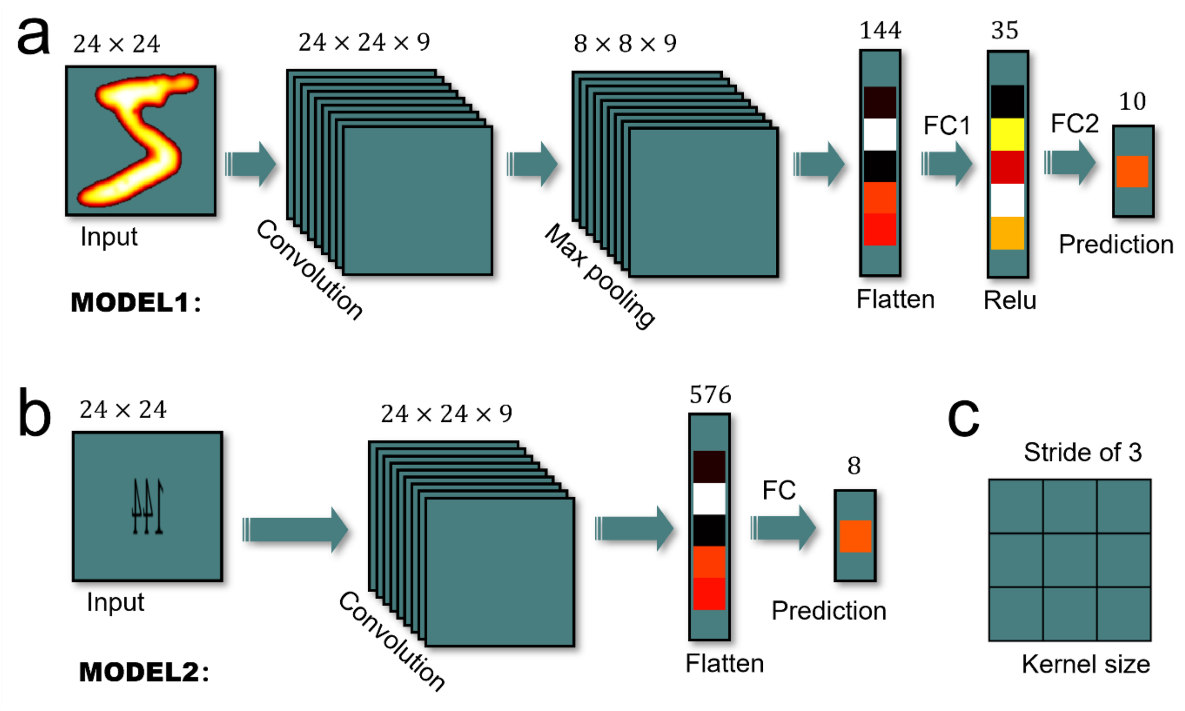

Figure S4. The digital neural network architecture used in the manuscript. (a) The neural network for amplitude-only object recognition. (b) The neural network process for polarized object classification. The label above indicates the input neuron numbers of each layer. (c) The size and stride number of kernel for convolution.

The digital neural network architectures for digit and polarization state classification described in the manuscript are shown in Fig.S4. We employ shallow neural network architectures with minimal numbers of digital layers. In both architectures ~50% of the overall FLOPs are moved into the optical layer. Model1 was used for the amplitude-only object recognition, while Model2 was applied for multi-functional (amplitude and polarization) classification. The FLOPs of each layer in the neural network were calculated as the following,

MODEL1:

$$\text{Convolution: FLOPs} = 2 \times \text{Channel} \times \text{Kernel shape} \times \text{Output shape} = 10368$$

$$\text{Max pooling: FLOPs} = (\text{Height} / \text{Stride}) \times \text{Depth} \times (\text{Width} / \text{Stride}) = 144$$

$$\text{FC1: FLOPs} = 2 \times \text{Input size} \times \text{Output size} = 10080$$

$$\text{FC2: FLOPs} = 2 \times \text{Input size} \times \text{Output size} = 700$$

MODEL2:

Convolution: $\text{FLOPs} = 2 \times \text{Channel} \times \text{Kernel shape} \times \text{Output shape} = 10368$

FC: $\text{FLOPs} = 2 \times \text{Input size} \times \text{Output size} = 9216$

S5: Measurement setup

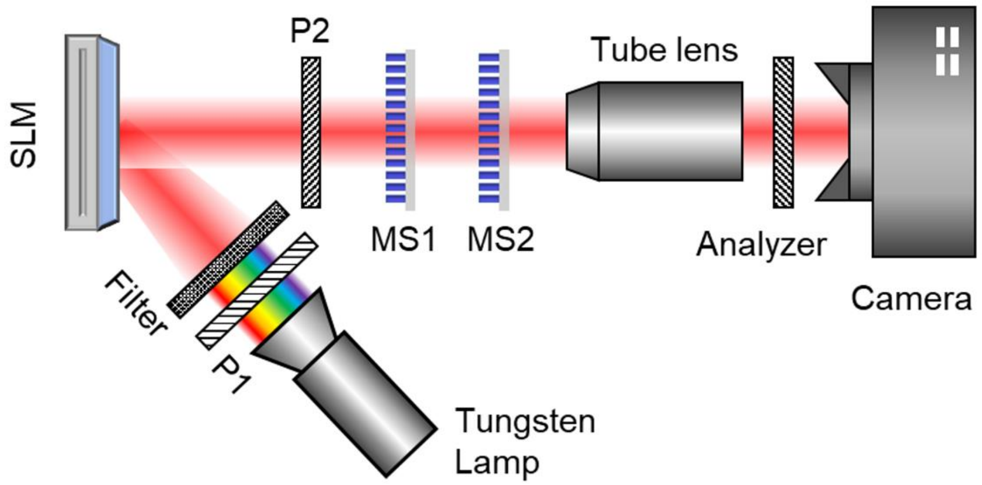

Figure S5. The characterization setup of optical neural network. P means the polarizer. MS is the metausrface.

S6: Comparison of kernels in optical and digital neural networks

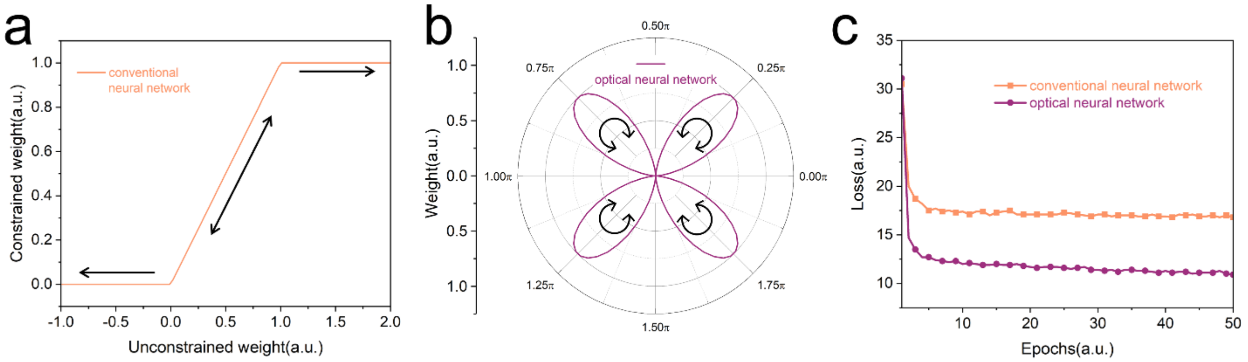

Figure S6. Comparison between conventional and optical neural networks. (a) and (b) The weight evolution for conventional and optical neural networks, respectively. (c) The loss evolution for conventional and optical neural networks as a function of training epochs.

In the manuscript, we used end-to-end optimization to train the hybrid neural network, where the data passing through the neural network (NN) obeys the physical transmission of the metasurface, modeled using the Jones matrix. On the other hand, in the training of a conventional neural network, the weight of each neuron will take an unbounded positive or negative value. To compare the training time, we restrict the weights of both the conventional and hybrid neural networks to be restricted to 0 to 1 since the optical signal cannot be negative and transmission cannot be above 1. In the case of the conventional network, we thus clamp the weights as shown in Fig.S6 (a), which leads to discontinuous weight iteration. On the other hand, the hybrid neural network has a weight dictated by sin $(2\theta)^2$, where θ is the rotation angle of the metasurface unit cell. In this case, the weight of each neuron is still restricted from 0 to 1 but the evolution of each weight is continuous during optimization.

In the hybrid neural networks, the continuous evolution of kernel weights based on a physical equation gives a more reasonable weight distribution as well as a better ability to avoid local minimum during optimization. Here, we did a comparison experiment, where two neural networks with the same architecture as well as initial states were trained based on the same dataset. One the of neural networks has the weights restricted by a built-in clamp function as shown in Fig.S6 (a) while the other has the constraints from Jones matrix in Fig.S6 (b). The latter restriction, with continuous weight evolution, provides convergence to a more accurate solution as shown in Fig.S6 (c).

S7: Verifying the significance of optical convolution layer

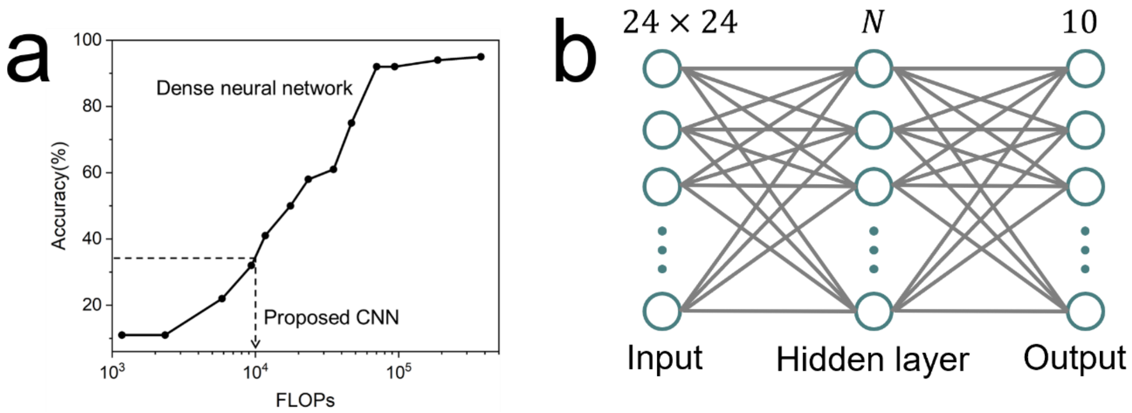

Figure S7. Performance diagram of dense neural networks (DNN). (a) The classification accuracy of DNN as a function of the FLOPs. (b) The architecture of testing DNN in (a).

The significance of optical convolution layer can be verified by two types of reference tests. For the first test, we fixed all the convolution kernel values at 1, demonstrating the capacity of the digital fully connected layer, since no feature map was generated from the convolution layer. In this case, the final accuracy converged to 79.2%. The second benchmark employed a random valued kernel. In this case, 79.7% overall accuracy was achieved. For comparison, 94.7% accuracy was achieved in the manuscript using the optimized optical convolution layer.

We further performed another testing without the convolution operations in order to show the necessity of kernels' modulation. In this case, the overall model was converted into a dense neural network (DNN). For the fair comparison, we calculated the classification accuracy as a function of FLOPs shown in Fig.S7 (a). The testing DNN architecture is shown in Fig.S7 (b), where a 2-layer DNN was used as same as the FC layer in the manuscript. The FLOPs were calculated by the following equation:

$$FLOPs = 2 \times (576 \times N + N \times 10) \qquad \text{(S6)}$$

Where N is the neuron numbers of the hidden layer. As increase of the neuron numbers, the rising FLOPs improved the complexity of NN, resulting in better performance and robustness. However, without convolution operations, the DNN requires more neurons (10 × more operations) to achieve a comparable performance (94%) with CNN as shown in Fig.S7 (a).

S8: Complex-amplitude modulation to reduce stride number

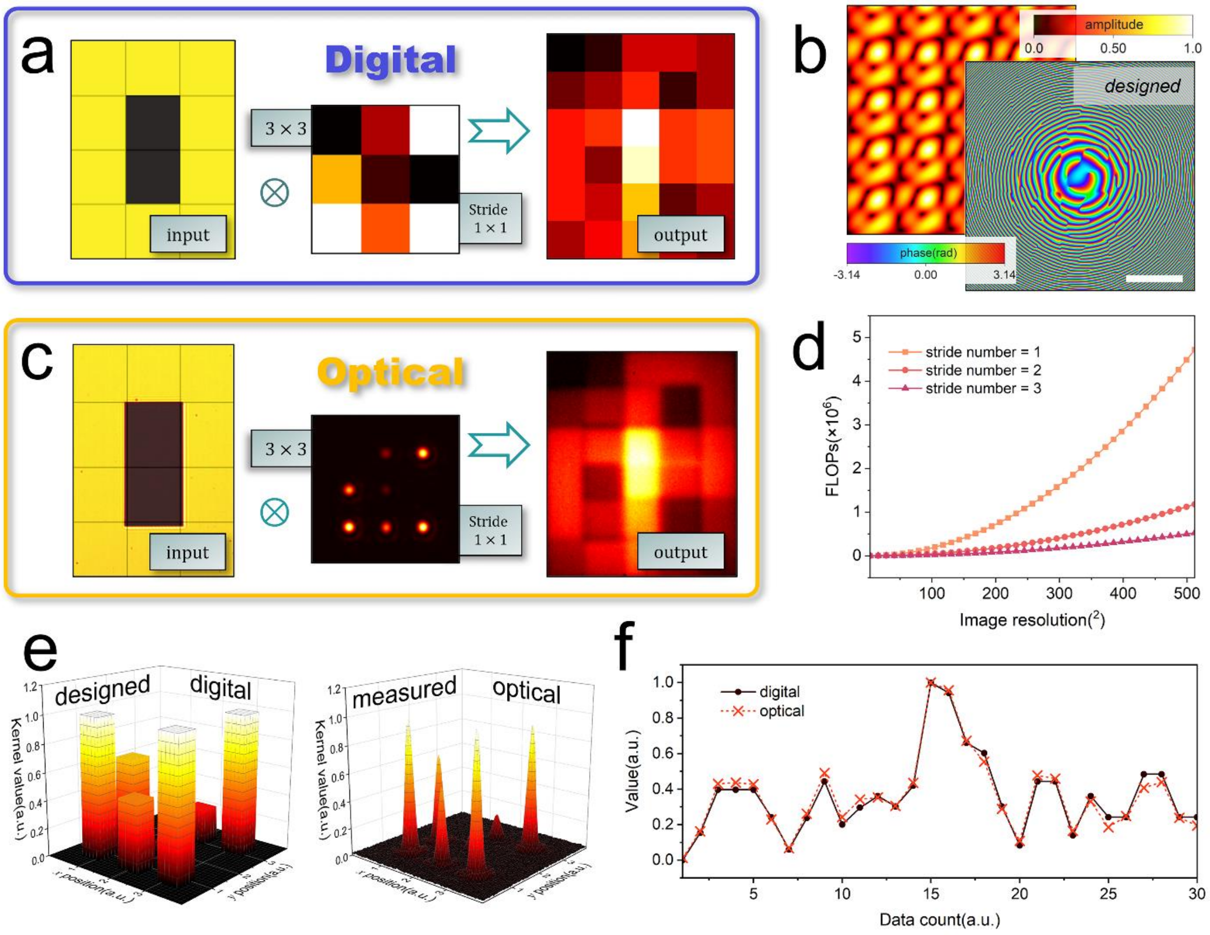

Figure S8. Complex-amplitude modulation method for low stride number convolution. (a) The digital convolution process with a designed kernel. (b) The designed complex-amplitude profile to operate optical convolution. (c) The convolution process performed by optical modulation. All data in (c) was optical measured based on fabricated devices. (d) The FLOPS of convolution as the function of stride number as well as the input image resolution. (e) The values of designed digital and measured optical kernels, respectively. (f) The comparison of the convolution results by the digital and optical operation.

The complexity of optical convolution can be improved by reducing the stride number, which can increase the robustness of the neural network. The reduced stride number (stride number less than kernel size) requires entanglement of the neurons, necessitating complex-amplitude modulation in the kernel layer. In this case, the metasurface-based kernel layer should be separated from the photodetector plane by a certain distance, such that the modulated signal can have interaction.

Fig.S8 (a) shows an example of digital convolution, where a 4×3 pixel array forms a simple image being convoluted with a 3×3 kernel. The stride number was set as 1, resulting in a

6×5 output image. To achieve this operation optically, we designed a single channel kernel layer with the complex-amplitude field based on the following equation,

$$F[\alpha(x,y),\phi(x,y)] = \sum_i^9 \exp\left[i \cdot \frac{2\pi}{\lambda}\left(f - \sqrt{f^2 + (x-a_i)^2 + (y-b_i)^2}\right)\right] \cdot w_i \qquad \text{(S7)}$$

where w_i represents each value in the kernel matrix, a_i and b_i correspond to the displacement of each unique focal spot from the center of the lens which dictates the stride number in the convolution process. The designed complex-amplitude field is shown if Fig.S8 (b). This modulation process is similar with the multi-channel metalens discussed in section S2, however, by controlling the displacement of each focal spot and transmission of each independent channel (the value of kernel pixel in this case), we can achieve optical convolution with stride number less than the kernel size. An optical measurement of this optical convolution process is exhibited in Fig.S8 (c) including the measured input image, focal spot profile, and convolution results, showing an excellent match with the digital operation result. The significance of small stride number is also shown in Fig.S8 (d). During the convolution process, with the same number of neurons, the complexity of the operations (FLOPs) is significantly increased when reducing stride number, leading to a more robust neural network.

In regards to the noise level in Fig.S8 (c), Fig.S8 (e) shows the values from the designed digital and measured optical kernels, respectively, while Fig.S8 (f) gives the comparison between the convolution results by the digital and optical operation. The data count represents the output values from Fig.S8 (a) and (b) after convolution and flattening. The maximum deviation, or noise level, is 7.6% demonstrating that optical convolution with stride of 1 can match well with the digital calculation.

S9: Complex polarization states classification

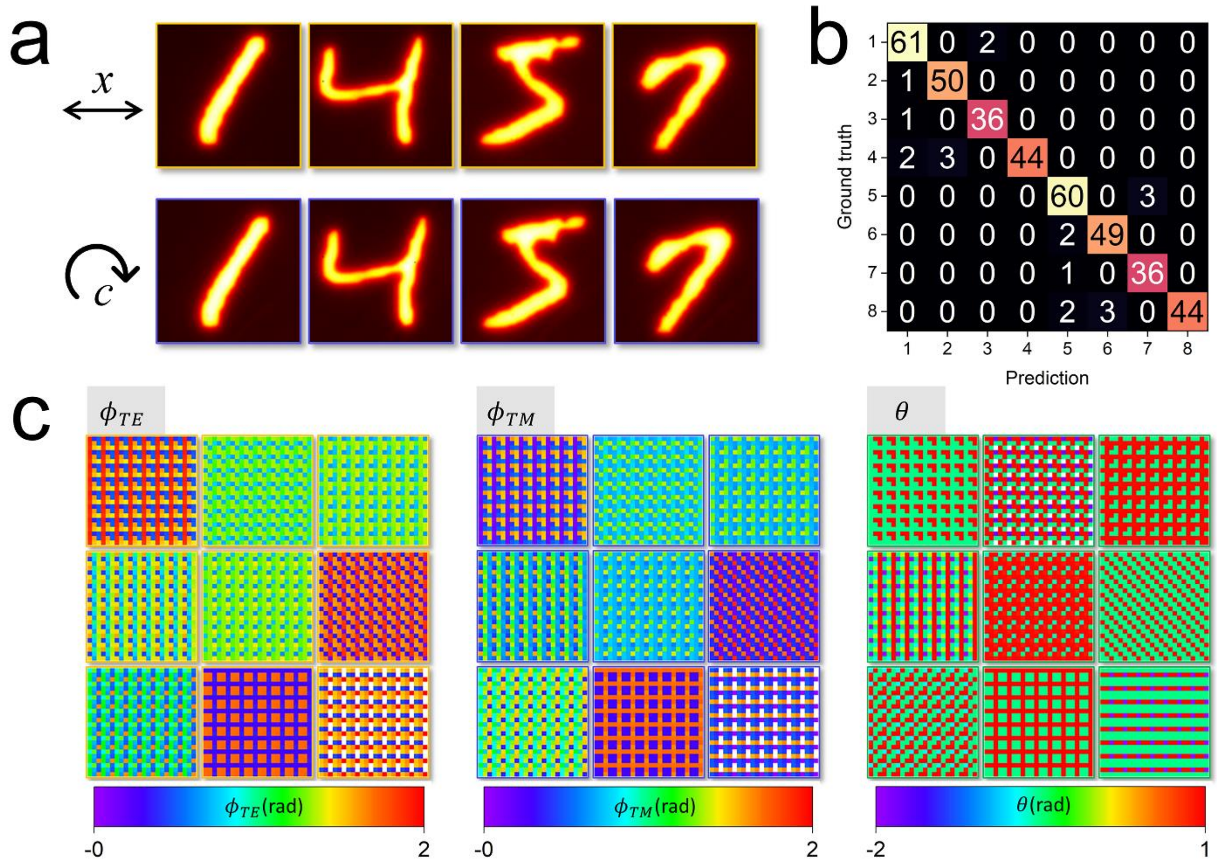

Figure S9. Demonstration of complex polarization states classification. (a) The data library is trained for the multifunctional neural network. The amplitude information contains four categories. Each category includes two different polarization states, one is linear and another is circular polarized. (b) The tested confusion matrix is based on the pre-trained neural network. (c) The pre-trained metasurface kernel layers are represented by the phase and rotation map.

Apart from spatial amplitude modulation, where the feature map was extracted for object classification, another unique capability of the metasurface is to convert the polarization state into a light intensity value. In this case, the output, polarization dependent, value is constant across the channel and is completely independent of the spatially varying amplitude value. Thus, the mechanism to distinguish polarization states is different from the object classification, which is based on the feature map analysis. Hence, both of these functions can be integrated into a single meta-optic for multifunctional analysis, which is one of the main advantages of the metasurface system.

To verify our proposed method can recognize a more complex situation, where the polarization states are not orthogonal, we employed four different amplitude objects with each object having two different polarization states - linear and circularly polarized. The confusion matrix is presented in Fig.S9 (b) and demonstrates 95% accuracy. Fig.S9 (c) also gives the pre-trained parameters of the metasurface kernel layer containing the phase and rotation map. It is worth mentioning that in the confusion matrix, the neural network system has 100% accuracy on polarization state recognition.

S10: Comparison between hybrid and diffractive neural networks

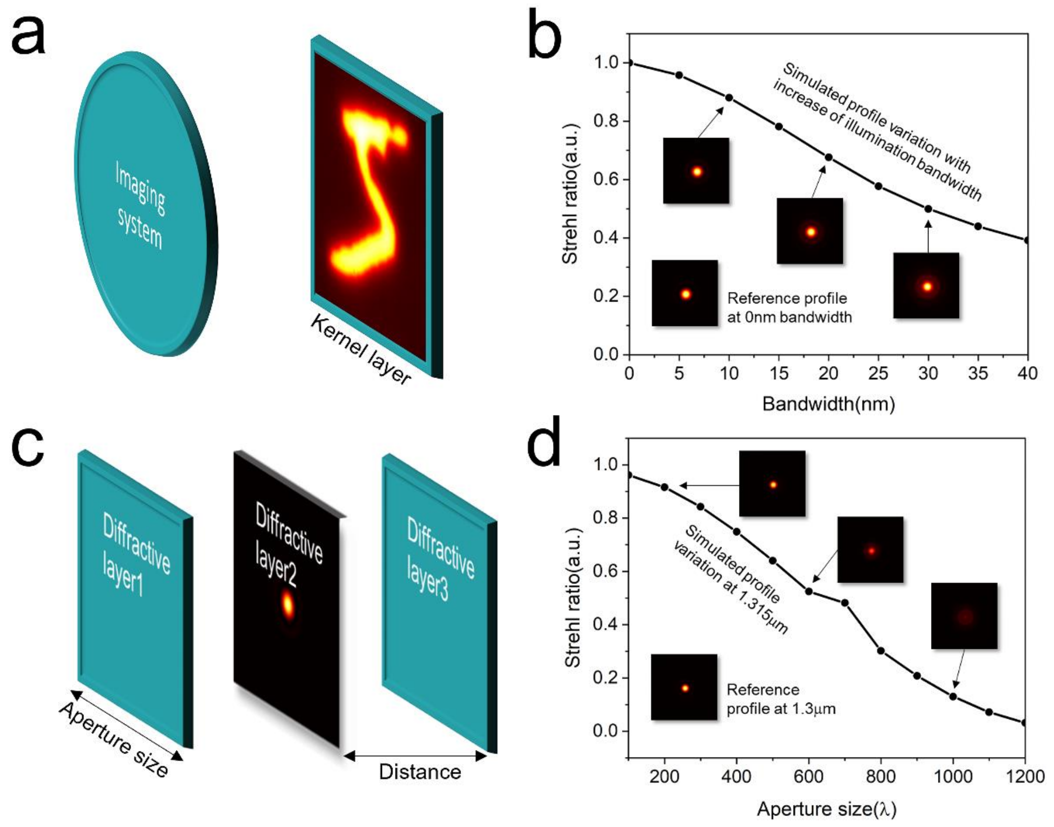

Figure S10. Bandwidth verification of meta-optic system and comparison to diffractive neural networks. (a) A schematic of proposed hybrid neural network proposed in manuscript. (b) The Strehl of the imaging system in terms of the illumination bandwidth. Insets shows point spread function (PSF) of imaging system as a function of the illumination bandwidth. (c) A schematic of a simple diffractive neural network. (d) The performance of diffractive layer as a function of input aperture size.

In the manuscript we used a bandpass spectral filter in front of a tungsten lamp as the illumination source to verify that our system works under incoherent light illumination. The filter bandwidth was 30nm. The bandwidth of the system is limited by the multi-channel lens since the kernel layer is broadband, as shown in the manuscript. We calculated the performance of the metalens based on the illumination bandwidth, as shown in Fig.S10 (a). Here, the calculation is based on the angular spectrum propagation method, where the point spread function of a metalens was calculated under illumination of various bandwidths, with each wavelength being equally weighted. It is worth mentioning that the diameter of our metalens is fixed at 2.4mm with an *f*/# of *f*/3 and moving to a smaller aperture or larger *f*/# would increase bandwidth. In Fig.S10 (b) we present the Strehl ratio of the lens for different illumination bandwidths, where a ratio of ~0.5 was achieved using the present configuration. Importantly, while the Strehl number is reduced with

increased bandwidth, the end-to-end design method mitigates aberrations as they are taken into account in the training/design. Thus, it is likely possible to further increase bandwidth.

Besides, there are several advantages of a discrete kernel neural network as described below. First, a discrete kernel allows for a memory efficient system architecture. Ideally, the neural network will have as few trainable parameters as possible. However, if arbitrary kernels (formed by a diffractive layer) are used, the system needs to design the phase modulation at each neuron to form the diffractive layer, which will drastically increase the data volume as the aperture size increases. Secondly, discrete kernels are needed if the system is dynamically reconfigured. Even in the case of a diffractive network, pixelization will be needed, due to practical limits on control electronics, to dynamically control the pattern of the metasurface. Furthermore, as outlined in the manuscript, as the size of the metasurface pixels is reduced their transmission will deviate from the local phase approximation also placing a limit on the practical size of reconfigurable metasurface pixels.

Compared to a diffractive neural network, the advantage of our proposed method can also be described in the following aspects. First, the proposed system has alignment tolerance due to end-to-end optimization. We incorporate 10% noise fluctuation and $\pm 3°$ rotation error in the training process, which makes the system less sensitive to the image position. However, the multilayer diffractive structure has a high sensitivity to the interlayer alignment process. According to our previous research as well as recent publications from other groups(53), (54) misalignment of even a single unit cell will lead to a large difference in the far-field intensity distribution, even for a simple 2-layer system, making it difficult to scale a high quality DNN into the visible spectrum. Secondly, our proposed method can be employed with broadband illumination due to the end-to-end optimization process, while a diffractive neural network, especially for a large aperture, will only work under narrow band (laser) illumination. To quantitatively demonstrate this, we calculate the performance of a simple DNN under different illumination bandwidths, as shown in Fig.S10 (c). In this architecture, the first layer will provide a specific neural weight, which will converge all the intensity into a spot, identical to a lens, hence we can use Strehl ratio to account for the performance. During the calculation, the *f*/# of the diffractive system was fixed as 2 for each layer and the system was designed at a wavelength of 1.3µm. Fig.S10 (d) shows the performance of the corresponding system working at a wavelength 15nm from the designed

wavelength (1.315um) as a function of aperture size. With increase in aperture size, the performance will deteriorate drastically, making a DNN unsuitable for broadband illumination at practical aperture sizes.
